# The Role of Symbiont Genetic Distance and Potential Adaptability in Host Preference Towards *Pseudonocardia* Symbionts in *Acromyrmex* Leaf-Cutting Ants

**DOI:** 10.1673/031.011.12001

**Published:** 2011-09-17

**Authors:** Michael Poulsen, Janielle Maynard, Damien L Roland, Cameron R Currie

**Affiliations:** ^1^ Department of Bacteriology, University of Wisconsin, Madison, 6145 Microbial Sciences Building, 1550 Linden Drive, Madison, WI 53706, USA; ^2^ Section for Ecology and Evolution, Department of Biology, University of Copenhagen, Universitetsparken 15, 2100 Copenhagen, Denmark; ^3^ Baylor College of Medicine, One Baylor Plaza, BCM215 Houston, TX 77030; ^4^ University of Houston-Downtown, One Main Street, Houston, TX 77002

**Keywords:** Actinobacteria, Escovopsis, recognition, symbiont choice, symbiosis

## Abstract

Fungus-growing ants display symbiont preference in behavioral assays, both towards the fungus they cultivate for food and Actinobacteria they maintain on their cuticle for antibiotic production against parasites. These Actinobacteria, genus *Pseudonocardia* Henssen (Pseudonocardiacea: Actinomycetales), help defend the ants' fungal mutualist from specialized parasites. In *Acromyrmex* Mayr (Hymenoptera: Formicidae) leaf-cutting ants, individual colonies maintain either a single or a few strains of *Pseudonocardia*, and the symbiont is primarily vertically transmitted between generations by colony-founding queens. A recent report found that *Acromyrmex* workers are able to differentiate between their native *Pseudonocardia* strain and non-native strains isolated from sympatric or allopatric *Acromyrmex* species, and show preference for their native strain. Here we explore worker preference when presented with two non-native strains, elucidating the role of genetic distance on preference between strains and *Pseudonocardia* origin. Our findings suggest that ants tend to prefer bacteria more closely related to their native bacterium and that genetic similarity is probably more important than whether symbionts are ant-associated or free-living. Preliminary findings suggest that when continued exposure to a novel *Pseudonocardia* strain occurs, ant symbiont preference is potentially adaptable, with colonies apparently being able to alter symbiont preference over time. These findings are discussed in relation to the role of adaptive recognition, potential ecological flexibility in symbiont preference, and more broadly, in relation to self versus non-self recognition.

## Introduction

Fungiculture in attine ants originated approximately 50 million years ago (Schultz and Brady 2008), and all 234 described attine ant species in 12 genera depend on fungi (Agaricales: Agaricaceae and Pterulaceae) for food (Chapela et al. 1994; Munkacsi et al. 2004). The ants provide substrate for cultivar growth and protection through removal of both competing and pathogenic microbes (Quinlan and Cherrett 1977; Currie and Stuart 2001). Only one specialized parasite of the ants' cultivar has so far been established; a microfungal parasite in the genus *Escovopsis* (Ascomycota: Hypocreales) (Currie et al. 1999a; Reynolds and Currie 2004). In addition to behavioral defenses against *Escovopsis* (Currie and Stuart 2001; Fernandez-Marin et al. 2006; Abramowski et al. 2011), attine ants engage in a mutualism with *Pseudonocardia* bacteria Henssen (Pseudonocardiacea: Actinomycetales) (Cafaro and Currie 2005), which produce antibiotics that are active against *Escovopsis* (Currie et al. 1999b; Poulsen et al. 2010; Cafaro et al. 2011). In many genera of attine ants, *Pseudonocardia* are housed on the cuticle of workers in elaborate structures, linked to exocrine glands (Currie et al. 2006). Callow workers eclose *Pseudonocardia-free* and obtain bacteria inocula on the cuticle 3–5 days post-eclosion, likely from colony members carrying a full or partial cover of the bacteria. The tight cuticular association with *Pseudonocardia*, in combination with molecular phylogenetic studies (Cafaro et al. 2011), suggests that fungus-growing ants have employed *Pseudonocardia* symbionts to deal with the garden parasite from the earliest stages of the ant-fungus mutualism (Poulsen and Currie 2006; Cafaro et al. 2011).

The ant-fungus-parasite-bacterium association appears to have undergone diffuse coevolution since the origin of fungiculture in ants (Mueller et al. 1998; Currie et al. 2003; Cafaro et al. 2011). Although broad patterns of specificity in the ant-bacterium association exist, strict cocladogenesis between the ants and the bacteria is disrupted. This is especially prevalent at the finer phylogenetic levels by *Pseudonocardia* strains switching between attine ant species and genera (Poulsen et al. 2005; Cafaro et al. 2011). The underlying mechanisms for such switching remain unknown, but have been predicted to lead to potential introduction of additional symbiont strains. However, within-colony diversity of *Pseudonocardia* appears low. Poulsen et al. (2005) found that 34 colonies of *Acromyrmex octospinosus* and *A. echinatior* each associate with a single strain, and Haeder et al. (2009) found a single *Pseudonocardia* strain within three colonies and two closely related distinct strains present in one *A. octospinosus* colony. This low within-colony *Pseudonocardia* diversity is predicted to help stabilize the association by reducing between-strain competition (Frank 1996, 2003; cf. Poulsen et al. 2007), and has been proposed to be antmediated (Zhang et al. 2007). In a recent report, Zhang et al. (2007) conducted behavioral experiments and showed that *Acromyrmex* workers are able to differentiate between their native and a foreign *Pseudonocardia* strain, and proposed that this could explain the potential exclusion of additional *Pseudonocardia* strains. However, the *Zhang* et al. (2007) study focused on preference between native versus non-native strains but did not elucidate ant preference when workers are challenged with two nonnative *Pseudonocardia* strains.

**Figure 1.**
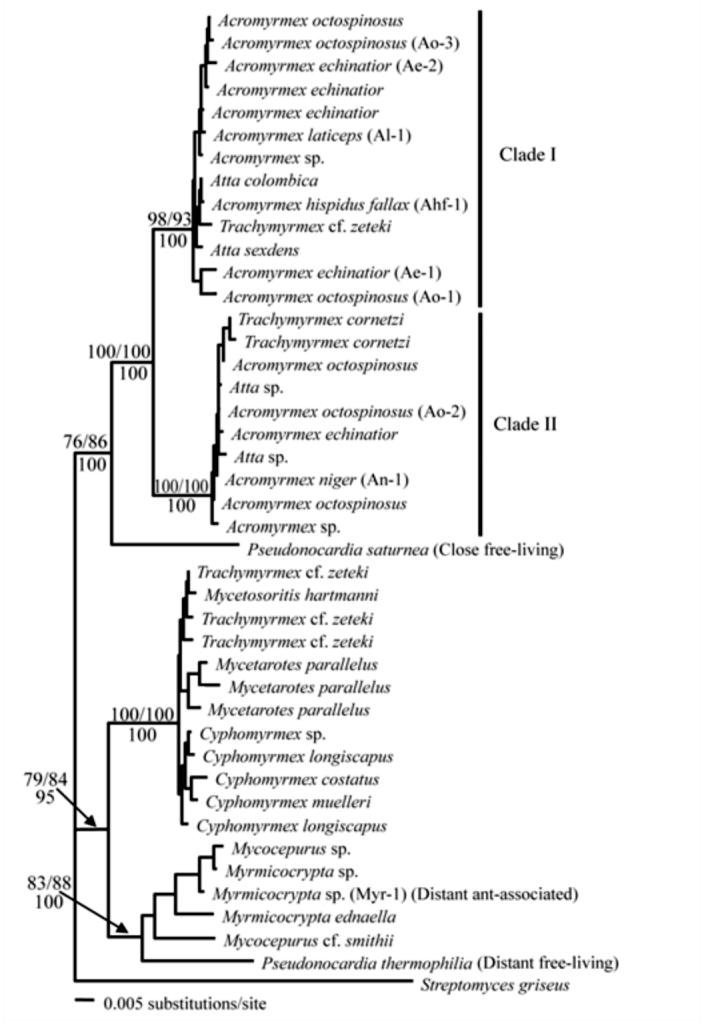
The phylogenetic placement of the strains associated with the colonies used in this study. The phylogeny is based on previously published partial sequences of 16S rDNA and nuclear Elongation Factor Tu (Zhang et al. 2007; Poulsen et al. 2007; Cafaro et al. 2011). The two clades of *Pseudonocardia* associated with *Acromyrmex* ants are highlighted (Clade I and II), as are the *Acromyrmex* colonies involved (codes in brackets), the closely (*P. saturnae*) and distantly (*P. thermophilia*) related free-living *Pseudonocardia*, and the distantly related ant-associated *Pseudonocardia*. Bootstrap support for branches are: 100 pseudo-replicates under Maximum Likelihood conditions (top right), 1000 pseudo-replicates under Maximum Parsimony conditions (top left), and 1000 pseudo-replicates under Neighbor-Joining conditions (bottom). High quality figures are available online.

This study incorporates behavioral preference assays developed by Zhang et al. (2007) to explore whether symbiont preference in *Acromyrmex* is affected by i) genetic distance between *Pseudonocardia* strains and ii) whether strains are ant-associated or freeliving *Pseudonocardia.* These questions are approachable in *Acromyrmex* Mayr (Hymenoptera: Formicidae), because i) ant species within this genus associate with two distinct phylogenetic clades of *Pseudonocardia* and ii) free-living *Pseudonocardia* are interspersed in the phylogeny of ant-associated *Pseudonocardia* (Cafaro et al. 2011; Figure 1). Although there were some interesting exceptions, our findings suggest that *Acromyrmex* ants tend to prefer bacteria more closely related to their resident bacterium, that host ant origin does not appear to play a strong role in symbiont preference, and that genetic distance is likely more important than whether symbionts are antassociated or free-living. We also explore whether symbiont preference can be altered after extended exposure to a novel *Pseudonocardia* strain, and our findings suggest that this is indeed possible.

## Materials and Methods

### Symbiont preference assay

The symbiont preference assay followed that of Zhang et al. (2007), wherein *Pseudonocardia* preference of workers was evaluated with workers choosing between their own and a novel bacterium. Here, the setup was modified so that ants were given a choice between two novel bacteria. Ants were briefly introduced to two fungus fragments, each 100–150 mg, from their resident colony. Each fungus fragment was inoculated with an aqueous suspension of bacteria prepared from pure bacteria cultures, and all inoculations were done blindly to avoid any potential observer bias. Immediately after inoculations, one ant was placed in a Petri dish. *Pseudonocardia* strain preference was scored by observation of the ant handling one of the fungus fragments for more than 10 seconds. For each choice combination, a total of 10 replicate Petri dishes were prepared with fungus fragments and bacterial inocula. Ants were tested one at a time and a total of 10 randomly picked ants were tested per dish, leading to a total of 100 ants tested per colony per choice combination (cf. Zhang et al. 2007). The labor-intensive nature of the setups did not allow for new Petri dishes to be used for individual ants. The Petri dish area not harboring fungus material was thoroughly wiped with a moist tissue between individual ant trials in an attempt to reduce possible effects of trace pheromones left by previous workers, with the assumptions that these compounds are water soluble and not sticky. In order to assure that preference between identical fungus fragments with the same inocula was random, 100 workers were distributed on 10 Petri dishes containing fungus fragments with only water, paralleled with Petri dishes having bacteria inoculated. Preference in the trials with bacteria inoculated was only considered reliable when preference of fungus fragments in these wateronly assays was not significantly different from a 50:50 distribution in a χ^2^ test (data not shown).

Five *Acromyrmex* colonies were used: *A. niger* Smith colony CC030327-02, *A. laticeps* Emery colony UGM030330-04, *A. echinatior* Forel colony Ae292 and colony CC03121201, and *A. octospinosus* Reich colony ST040116-01 (Figure 1). All colonies were housed at the University of WisconsinMadison in plastic containers, and colonies were provided with maple or oak leaves three times per week. Due to the labor intensive nature of the trials, only 1–2 choice combinations for one or two colonies could be done per day; results presented here are consequently accumulated over the course of the summer 2007 and summer 2008.

Worker symbiont preference was explored between: i) *Pseudonocardia* from the same *Acromyrmex*-associated phylogenetic clade vs. *Pseudonocardia* from different *Acromyrmex*-associated phylogenetic clade; ii) *Escovopsis* from the same *Acromyrmex*associated phylogenetic clade vs. free-living *P. saturnae* (Hirsch); iii) *Pseudonocardia* from different *Acromyrmex*-associated phylogenetic clade vs. free-living *P. saturnae;* iv) free-living closely related *Pseudonocardia* (*P. saturnae*) vs. more distantly related freeliving *Pseudonocardia* (*P. thermophilia* Henssen); and v) distantly related antassociated *Pseudonocardia* from *Myrmicocrypta ednaella* Mann (Hymenoptera: Formicidae) vs. free-living more closely related *Pseudonocardia* (*P. saturnae*) (Table 1, 2, Figure 1). All bacteria involved (Table 1, 2) were obtained from previous studies (Poulsen et al. 2007; Zhang et al. 2007; Cafaro et al. 2011), and all bacterial sequences are available in GenBank. The results of the symbiont preference behavioral experiments were evaluated using χ^2^ test of distribution of preference being different from 50:50. The results are given in Table 3.

### Reversal experiment

**Figure 2.**
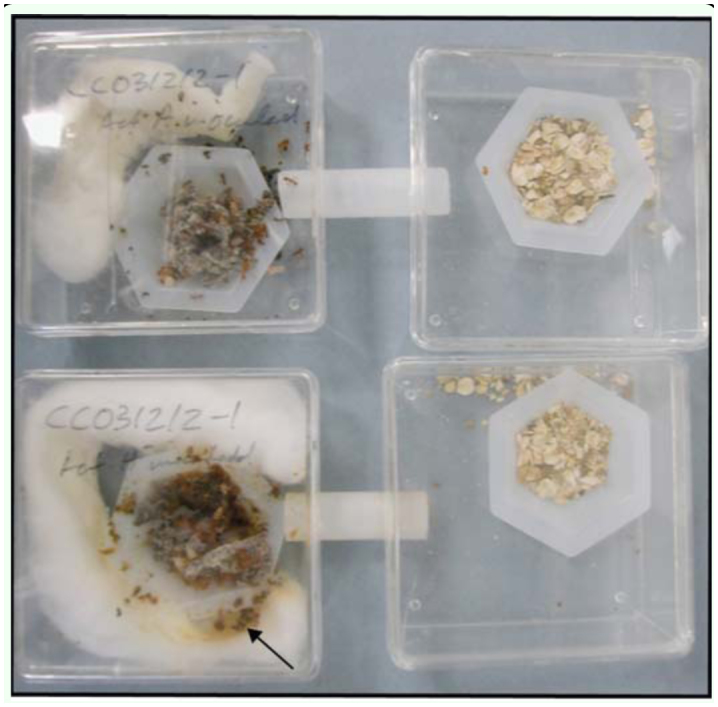
The setup for the reversal experiment: Individual dualchamber setups included two weigh boats, one with fungus garden (left) and one with oatmeal for food (right). A liquid suspension of *Pseudonocardia* in water was inoculated daily for four weeks to the fungus fragment, and either the resident bacterium (top image) or a novel bacterium (bottom image) was inoculated. Sub-colonies receiving their native *Pseudonocardia* rarely accumulated garbage material (top image), whereas sub-colonies that received a novel bacterium had an increase in the amount of accumulated garbage (brown material on the moist cotton (arrow), surrounding the weigh boat in the bottom picture) (see text for detail). High quality figures are available online.

A reversal experiment was conducted in order to determine if prolonged exposure to a nonpreferred bacterium altered ant preference. A total of six colonies from five *Acromyrmex* species (Table 4) were used. Dual chambers had a connecting tube (Figure 2). One chamber contained approximately 200 mg of fungus material, pupae, larvae, and minor workers; brood and minor workers are hard to remove without disrupting the fungus garden. These materials were placed in a weigh boat (38 mm/25 mm max diameter × 10 mm depth). A piece of moist cotton was placed around the dish to ensure high humidity within the chamber. The other chamber contained a weigh boat with oatmeal to provide a food source for the ants. Newly eclosed (callow) major workers not yet carrying *Pseudonocardia* on their cuticle (Poulsen et al. 2003) were subsequently introduced to the sub-colonies. Because the number of callow workers available varied between source colonies, the total number of callows available was split equally between control and treatment sub-colonies. Control sub-colonies were inoculated with 100 µl of an aqueous suspension of their native bacterium daily for one month. In contrast, treatment sub-colonies received a non-native bacterium, previously determined to be distinct from the ants' native strain, within the other leaf-cutting ant-associated *Pseudonocardia* clade (cf. Figure 1). In order to assure relatively stable sub-colonies during the duration of the experiment, moist cotton and fungus fragments were replaced every fourth day. After the one-month exposure period, the *Pseudonocardia* preference of major workers was assessed using the same assay as described above in order to evaluate whether prolonged exposure to a novel bacterial strain would affect worker ant preference. The results of this preference assay were assessed using χ^2^ tests as described above.

## Results

### Symbiont preference assay

Table 3 summarizes the results of the symbiont preference assays. In the experiment comparing the preference of a *Pseudonocardia* strain from the same versus a novel phylogenetic clade (Figure 1), three colonies chose *Pseudonocardia* from their own clade (Table 3). In contrast, workers from one colony handled the fungus piece containing *Pseudonocardia* from the different clade significantly more frequently, and the remaining colony showed no preference between the two (Table 3). In the experiment where ants were given a choice between *P. saturnae* and a *Pseudonocardia* from their own clade, two of the five colonies chose the bacterium from their own clade, two colonies did not show a preference, and one colony chose *P. saturnae* (Table 3). In the experiment evaluating two bacterial strains that were relatively closely related but one being antassociated and one being free-living (*Pseudonocardia saturnae*), four of the five colonies did not show a preference, while workers from the remaining colony more frequently handled the fungus fragment inoculated with *Pseudonocardia saturnae* (Table 3). When given a choice between two free-living bacteria, a closely related strain (*P. saturnae*) and a more distantly related strain (*P. thermophilia*), three of the five colonies choose *Pseudonocardia saturnae*, while the remaining two colonies did not show a preference. In the final behavioral preference experiment in which ants were presented with a free-living (*P. saturnae*) and a more distantly related ant-associated (from *Myrmicocrypta* sp.) bacterium, two of the five colonies did not show a preference, while two colonies preferred the distant ant-associated bacteria and one colony preferred *P. saturnae*.

### Reversal experiment

The prediction for this experiment was that if continued exposure of a bacterium in fungus-growing ant colonies governs the *Pseudonocardia* preference in workers, inoculations of either the ants' native or a non-native bacterium for four weeks would alter their subsequent preference in choice assays. Four of the six colonies used in this experiment indeed displayed altered preference after exposure to a non-native *Pseudonocardia* strain compared to control colonies inoculated with their own strain. Three of these colonies significantly more frequently choose the new bacterium, while ants from the fourth colony most frequently handled the fungus fragment with their native strain (Table 4). The last two colonies showed no significant preference (Table 4). Interestingly, mortality of workers within sub-colonies was vastly different between control and treatment sub-colonies: although all colonies started with the same number of ants within the sub-colony, by the end of the experiment the six treatment sub-colonies contained between 13.5% and 53.3% (mean±SE: 35.1±5.6%) fewer live callow workers compared to their respective control sub-colonies (Table 4). Furthermore, it appeared that all treatment sub-colonies removed more garden debris material than the controls (Figure 2; MP, DR, personal observations).

## Discussion

Our symbiont preference assays confirm previous findings that *Acromyrmex* leaf-cutting ants can recognize different *Pseudonocardia* strains (Zhang et al. 2007). Although variable across colonies, our results show that workers tend to prefer *Pseudonocardia* strains more closely related to their own, and preference was generally less apparent if the bacteria were more closely related to each other than to the ants' own bacterium. This was the case: when ants were presented with a bacterium from their own clade versus that of a different clade (3 of 5 colonies); when ants were presented with a bacterium from their own clade versus *P. saturnae* (2 of 5 colonies); and when ants chose between two free-living bacteria (3 of 5 colonies) (Table 3). This suggests that similarity to the ants' own bacterium plays a role in preference, and that preference for a closely related bacterium is upheld even if this bacterium is free-living. More specifically, when presented with two relatively closely related bacterial strains—one free-living and one from a different ant-associated clade— four of five colonies showed no preference (Table 3). The finding that ants prefer bacteria closely related to their native strain suggests that switches, which are known to occur in population-level studies (Poulsen et al. 2005; Mikheyev et al. 2008; Cafaro et al. 2011), are most likely to be towards a bacterium closely related to the resident bacterium. Although our findings do not unambiguously show these preferences, they do suggest that switches would not be restricted to non-native ant-associated *Pseudonocardia* strains. This is perhaps especially so because in most preference assays we examined preference towards *Pseudonocardia* from allospecific colonies, and occasionally colonies from distant geographic origins. Ideally, future studies should include more detailed investigations between colonies from both within and between individual *Acromyrmex* species and their geographic origins. However, we cannot rule out that ant preferences are governed by interactions between the bacteria inoculated in the fungus fragment and the fungus strain; future studies that can elucidate the role of such potential bacteria-fungus interactions in host ant preference are desirable.

The reversal experiment indicated that symbiont preference can be altered (Table 4). Ants from four out of six colonies changed their preference after four weeks of exposure, supporting that the ‘resident’ bacterium is recognized, but that prolonged exposure to another bacterium can alter this preference. In three of these four colonies, the preference was changed to the novel bacterium, suggesting adaptive recognition and acceptance of this novel bacterial strain. Except in one instance, control sub-colonies in which ants were exposed to their own bacterium for four weeks did not significantly prefer their own bacterium to a non-native strain (Table 4), contrary to expectations from Zhang et al. (2007). This suggests that more precise methods for determining ant preference are needed to fully establish the role of exposure to a novel bacterium. More effective methods to expose the ants to the bacteria could involve longer exposure time and/or a better preference assay. It remains unclear if willingness of workers to accept a non-native strain increases if the workers have lost their native bacterium. We would predict that this would be the case if successful reestablishment of the symbiosis would represent a selective advantage for the ants in the defense against *Escovopsis* (cf. Poulsen et al. 2010).

Interestingly, all six colonies inoculated with a novel bacterium experienced increased worker mortality and increased accumulation of garbage material compared to control subcolonies receiving their own bacterium. This indicates that colonies with a fungus garden inoculated with a novel bacterium did worse than when the resident bacterium was inoculated. The reason for increased mortality is unclear, while the removal of non-native material within fungus gardens with subsequent garbage accumulation is analogous to the removal of non-native fungus clones (Bot et al. 2001), pathogens, and parasites (Currie and Stuart 2001; Abramowski et al. 2011). This supports the idea that the ants distinguish their own bacteria from novel bacteria, and further suggests the active suppression of non-native *Pseudonocardia* bacteria. Whether similar responses are present towards other bacterial symbionts recently recognized in fungus-growing ants (Pinto-Tomás et al. 2009; Scott et al. 2010; Suen et al. 2010) remain to be investigated.

The findings presented in this paper support the hypothesis that leaf-cutting ants in the genus *Acromyrmex* are able to recognize different *Pseudonocardia* strains from each other, and that when workers are presented with two non-native bacterial strains, they prefer bacteria more closely related to their native strain. Further, the adjustment in preference may imply selective advantage for leaf-cutting ants if this allows for the acquisition of novel bacterial strains with novel metabolic capacities (cf. Zhang et al. 2007; Cafaro et al. 2011). It also suggests that preference is adaptive and analogous to ant preference for the cultivar fungus, which in a similar manner can be altered by continuous exposure to a novel fungal strain (Bot et al. 2001; Mueller et al. 2004; Poulsen and Boomsma 2005). The mechanism of mutualistic fungus recognition has been proposed to be due to chemical compounds produced by the fungus itself (Richard et al. 2007) and compounds transferred from the ant brood (Viana et al. 2001), resulting in the fungus garden obtaining a colony-specific odor. In contrast, the mechanism of bacterial recognition is unknown, but is most likely caused by unknown bacteria-specific odors that are recognizable by the ants. The finding that symbiont acceptance by the ants can be altered after exposure to a novel symbiont in both suggests that adaptability in preference is similar between the two symbionts. Such recognition and adaptability is thus likely to be analogous to self versus non-self recognition, with acceptance of self (i.e., native symbionts) and antagonism towards, or removal of, non-self (i.e., non-native symbionts).

Self-recognition and antagonism towards nonself is a crucial component of social insect biology, where recognition of, and response to, colony members and individuals from other nests are governed by colony members learning and constantly updating their reference of self from the collective colony odor (cf. Boulay et al. 2000; Liang and Silverman 2000). This is largely analogous to immune system responses, in which non-self components within an insect or vertebrate body are targeted and suppressed or removed (Cremer and Sixt 2009; for review see Boehm 2006). However, because many non-self components are beneficial (i.e., mutualistic and commensal symbionts), mechanisms that allow for the immune system to distinguish between dangerous and beneficial non-self are crucial (cf. Cremer and Sixt 2009). Similar processes of recognition and adaptive change in preference of mutualistic symbionts are likely to govern the ant-fungus and ant-bacterium associations. Recognition of the resident symbiont in a successful association allows for the maintenance of host-symbiont homeostasis, thereby preventing the invasion of non-native symbiont strains (cf. Poulsen and Boomsma 2005; Poulsen et al. 2005). In contrast, the ability for adaptive change in preference may imply significant flexibility in a symbiotic association by allowing for the acquisition of novel symbiont strains either after symbiont loss (Adams et al. 2000; Poulsen et al. 2009) or after changes in abiotic factors that result in the maintenance of sub-optimal symbiont strains (cf. Mueller et al. 2004).

**Table 1.**
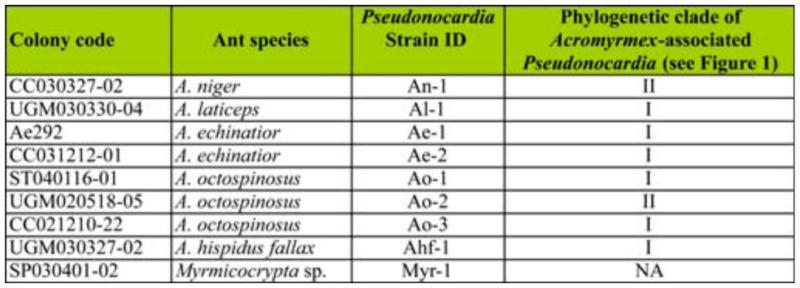
Colonies and *Pseudonocardia* strains involved in this study.

**Table 2.**
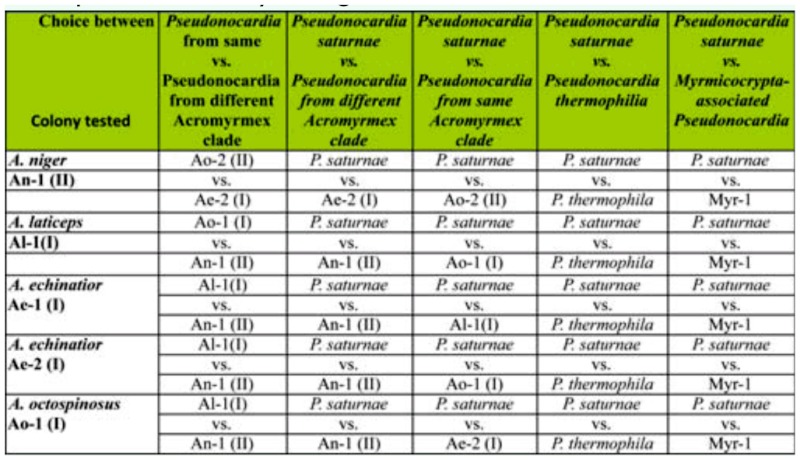
Colony origin of the bacterial strains involved in the behavioral preference assays. *P. saturnae* and *P. thermophilia* were obtained from the USA Agricultural Research Service (ARS) culture collection (Peoria, IL). The ant species name, *Pseudonocardia* strain ID (Table 1), and the phylogenetic clade (I or II, Figure 1) are given for each colony and strain involved in the tests. The results of the preference assays are given in Table 3.

**Table 3.**
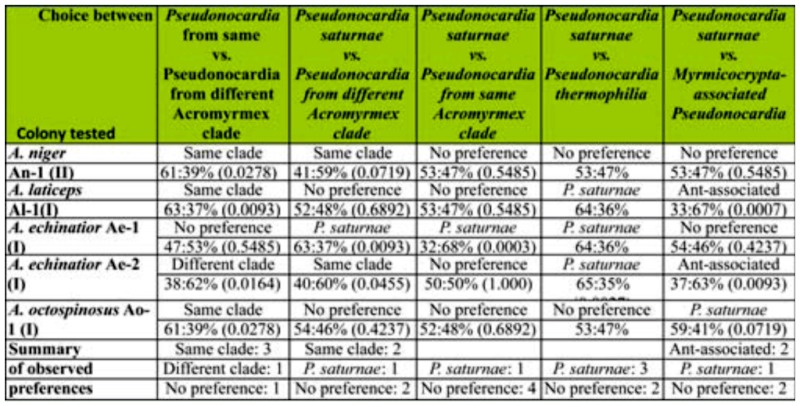
The results of the behavioral preference assays. The bacteria inoculated are given in Table 2, and for each colony tested, the ant species name, *Pseudonocardia* strain ID, and phylogenetic placement are given. The direction of preference for each combination for each colony is given, as are the proportions observed (below overall preference) and the *p*-value (in parenthesis) derived from a χ^2^ test of deviation from a 50:50 distribution (see text for details). N = 100 for all trials. The bottom row gives a summary of the observed preferences.

**Table 4.**
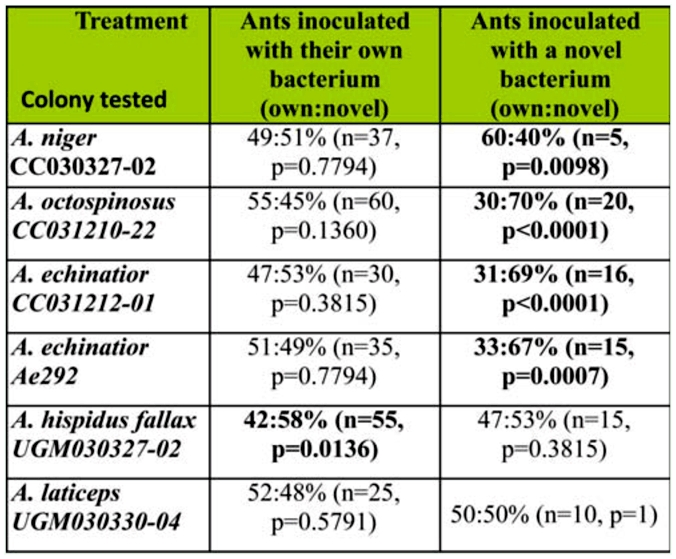
This table summarizes the results of the reversal subcolony experiment. Ants from the six colonies in the left column were subjected to either their own (middle column) or a novel bacterium (right column) for four weeks, after which their preference was evaluated in a preference assay testing ant preference of original verses novel bacterium. Because the number of ants present in sub-colonies after prolonged exposure varied, the exact number of ants tested in the subsequent preference assay is indicated for each of the tests. The distribution of preference was tested using a χ^2^ test, and preferences significantly different from a 50:50 distribution are highlighted in bold.
